# A scoping review on innovative methods for personality observation

**DOI:** 10.3389/fpsyg.2023.1112287

**Published:** 2023-03-08

**Authors:** Lucia Luciana Mosca, Grazia Isabella Continisio, Natascia De Lucia, Elena Gigante, Carmela Guerriera, Nelson Mauro Maldonato, Enrico Moretto, Ottavio Ragozzino, Veronica Rosa, Chiara Scognamiglio, Roberta Stanzione, Daniela Cantone

**Affiliations:** ^1^SiPGI - Postgraduate School of Integrated Gestalt Psychotherapy, Torre Annunziata, Italy; ^2^Department of Translational Medical Sciences, University of Naples Federico II, Naples, Italy; ^3^Department of Neurosciences and Reproductive and Odontostomatological Sciences, University of Naples Federico II, Naples, Italy; ^4^Department of Psychology, University of Campania, Luigi Vanvitelli, Caserta, Italy; ^5^ASPICARSA (Association of Applied Scientific Research ASPIC), Rome, Italy

**Keywords:** personality, unconventional study method’s, big five, personality profiles, scoping review

## Abstract

**Background:**

Personality’s investigation has always been characterized as a central area of research for psychology, such that it was established in the 1920s as an autonomous scientific-disciplinary field. Identifying and observing the people’s typical ways of “being in the world” has made possible to define the predictability of a pattern of behavioral responses related both to the possession of distinct characteristics of the agent subject and to specific environmental situations. In the actual scientific landscape, there is a strand of research that makes a description of personality through methodologies and indicators not usually used by psychology, but scientifically validated through standardized procedures. Such studies seem to be significantly increasing and reflect the emerging need to have to consider the human being in his or her complexity, whose existential and personal dimensions can no longer be traced to classification systems that are divorced from the epochal reference.

**Objective:**

In this review, attention is focused on highlighting publications in the literature that have included the use of unconventional methods in the study of nonpathological personality, based on the Big Five theoretical reference model. To better understand human nature, an alternative based on evolutionary and interpersonal theory is presented.

**Design:**

Online databases were used to identify papers published 2011–2022, from which we selected 18 publications from different resources, selected according to criteria established in advance and described in the text. A flow chart and a summary table of the articles consulted have been created.

**Results:**

The selected studies were grouped according to the particular method of investigation or description of personality used. Four broad thematic categories were identified: bodily and behavioral element; semantic analysis of the self-descriptions provided; integrated-type theoretical background; and use of machine learning methods. All articles refer to trait theory as the prevailing epistemological background.

**Conclusion:**

This review is presented as an initial attempt to survey the production in the literature with respect to the topic and its main purpose was to highlight how the use of observational models based on aspects previously considered as scientifically uninformative (body, linguistic expression, environment) with respect to personality analysis proves to be a valuable resource for drawing up more complete personality profiles that are able to capture more of the complexity of the person. What has emerged is a rapidly expanding field of study.

## Introduction

### Background

The personality’s investigation has always been characterized as a central field of research for psychology, such that it was established in the 1920s as an autonomous scientific-disciplinary field. Personality can be defined as the set of characteristics of the person, capable of producing congruent patterns of thinking, feeling and behaving and, in this sense, identifying and observing the people’s typical ways of “being in the world” (Biswanger, 1973) allowed in the course of the development of psychological science, and still allows today, to define the predictability of a behavioral pattern responses’ related both to the possession of specific characteristics of the subject agent and to specific environmental situations. In this sense, therefore, psychological theories have clarified the extent to which personality is the result of the complex interaction between biological factors (temperament) and environmental factors (character). Cloninger et al. (1993) first identified this interactive dichotomy between the biological and situational element of personality, and later Kagan (2011) clarified that temperament should be considered not as a constituent principle of a single personality type, but rather as a predisposition that tends to develop toward multiple possible profiles. In the timeline of psychological science development, we find that Kagan’s conception was preceded by investigations by various researches aimed to identifying the existence of factors that could group the extreme variability of the personality’s elements within common classes. Such studies produced descriptions of “personality types,” that are characteristics ways in which people organize their mental, behavioral and relational functioning, which refer to the possession of specific personality traits. Trait is conceived as an inclination to act in a not directly manner or exclusively related to changing circumstances and environments, so it presupposes the existence of a stable and enduring tendency to experience and regulate emotions, to process information and take action. Trait Theory was conceived by Allport (1937), who summarized, with this word, the existence of “personality fundamental units”, identified in about 4,000 of them, and distinguished into three types, cardinal central and secondary, according to their constituent characteristics. This theorization in the landscape of personality study was very important and stimulated the scientific interest of other researches who made subsequent modifications and reworkings to it, finally arriving at the theory of the “Big Five,” i.e., the theory of the five major personality factors that, in the actual psychology scientific landscape, is conceived as the one capable of explaining the greatest individual variability. This theory, elaborated subsequently by Costa and McCrae (1992), emphasizes the individual dispositional tendencies, which are described by the five dimensions that decipher the person’s specific behavior according to the particular combination of them. Currently, such a reading model has become the almost unambiguous reference in the field of personality science as the discovery of its presence in many different cultures has led to the understanding that the Big Five are attributes proper to human nature. For this reason, all studies and research about personality, and the possible combination of characteristics useful in defining profiles of personality, use precisely the Big Five model as the epistemological theoretical background of reference. Within the vast panorama of these studies lies an innovative strand of scientific research that arrives at a description of personality by resorting to the use of methodologies, as well as indicators, so far not usually used by psychology even if scientifically validated through standardized procedures. Such studies seem to be significantly increasing, reflecting the emerging need to have to consider the human being in his or her complexity, whose existential and personal dimensions can no longer be traced to classification systems that are divorced from the epochal reference. In fact, a first set of identified writings affirms the possibility of studying and describing the personality of individuals precisely from the consideration of the modern ecosystem in which human behavior takes place, in which it is no longer possible to disregard the use of information technology devices and the communicative and interaction potential they offer, through the various social media available. In such studies, through sophisticated semantic analyzes, significance is given to the linguistic expressions used by users both on social networks to share their moods, activities and thoughts, and in self-descriptions, considered in this sense as reliable indicators of personality style. Alongside this, an additional large pool of scientific production focuses attention on the observation of the body and its movements in space and the possession of certain morphological or expressive features as typical and characteristic of certain personality patterns. Finally, those studies that used an integrated theoretical approach as an epistemological backdrop for the observation and description of personality were selected as the last set: the use of such an approach is what best captures the complexity of human beings (Iennaco et al., 2019). From such studies, those using machine learning methods for personality research and assessment have been differentiated as a further considered category (Sperandeo et al., 2019).

### Aim

In this review, attention is focused on highlighting publications in the literature that have included the use of unconventional methods in the study of nonpathological personality, based on the Big Five theoretical reference model. The aim is to introduce a vision to understand personality in a broader way, taking into account indicators not usually included in standardized tests.

## Method

### Search and retrieval

Following the PRISMA Extension for Scoping Reviews (PRISMA-ScR) guidelines (Tricco et al., 2018), a scoping review of the literature was conducted in order to find articles on the topic published in peer-reviewed journals. It was decided to define a specific time frame, useful to focus on the most recent writings, in a period between January 2011 and January 2022. The surveys were conducted through two main search engines, such as PubMed and Google Scholar, choosing various keywords (listed in the Table 1), and the collected material from the two different repositories was then further examined to eliminate any duplicates and then unified into a single dataset.

**Table 1:** Keyword list.

Keyword
Personality
Computational methods
Big five
Personality traits
Personality assessment

The eligibility and exclusion criteria are summarized in Table 2.

**Table 2:** Eligibility and exclusion criteria.

Eligibility criteria	Exclusion criteria
1. Published in English	1. Books, editorial, opinion papers, literature reviews
2. Published in a peer-reviewed journal	2. Articles on the diagnosis of personality disorders
3. Articles on the description of non-pathological personality	3. The research did not really include the use of computational methods to analyze data.
4. Articles describing personality using unconventional methods	4. Data analysis was not suitable for the scoping review process.

### Article selection

The first phase of screening, carried out automatically by the system based on the keywords entered, resulted in the highlighting of more than 800 articles. In the next stage, all articles that, by title and abstract, seemed to fall within the objectives of the present work were extrapolated from that number. In this way, the number of selected articles was reduced to 200, and was equally distributed among the two independent researchers who participated in the selection process. From this initial number, after full text reading, 50 studies were distinguished, which, subjected to further skimming and based on the exclusion criteria listed above, were reduced to 36. Further analysis conducted later to identify those articles whose data were not useful to be processed according to the Prisma review method set the final number of included studies at 18.

The article selection process took into consideration the parameters developed by Ouzzani et al. (2016). Conflicts between researchers were resolved through mutual confrontation, providing for the possibility of the involvement of a third researcher in case of a lack of agreement.

### Assessment of methodological quality

The assessment of the methodological quality of the studies was not applied.

### Data extraction and selection

The review process used the collaboration of several experts: one in psychiatry and complex systems modeling, two in process research in psychotherapy, and one in psychology specializing in machine learning.

### Data charting process

The results are presented through tables, graphs, diagrams and narrative descriptions. The purpose of the tables, graphs and diagrams is to summarize the relevant data obtained, providing an overview of the various methods used.

### Critical evaluation of individual sources of evidence

Prior to inclusion, a process of critical selection of the sources consulted was carried out. In particular, the parameter of objectivity was considered (McMillan, 2001), i.e., the ability of the articles to highlight research methods and findings in the study of non-pathological personality through the use of unconventional methods of observation and assessment.

### Summary measures

Not applicable for scoping reviews.

### Risk of bias across studies

Not applicable for scoping reviews.

### Additional analyzes

Not applicable for scoping reviews.

### Results

#### Selection of sources of evidence

The final number of papers evaluated as eligible and thus included in the review was 18. In the various consecutive stages of the review process, the use of exclusion criteria first removed all papers whose abstracts showed little congruence with the topic; book chapters, previous reviews on the same topic, editorials and opinion papers, articles focused on the diagnosis of personality disorders, research using conventional methods for studying personality, and articles written in a language other than English were also excluded.

**Figure 1:**
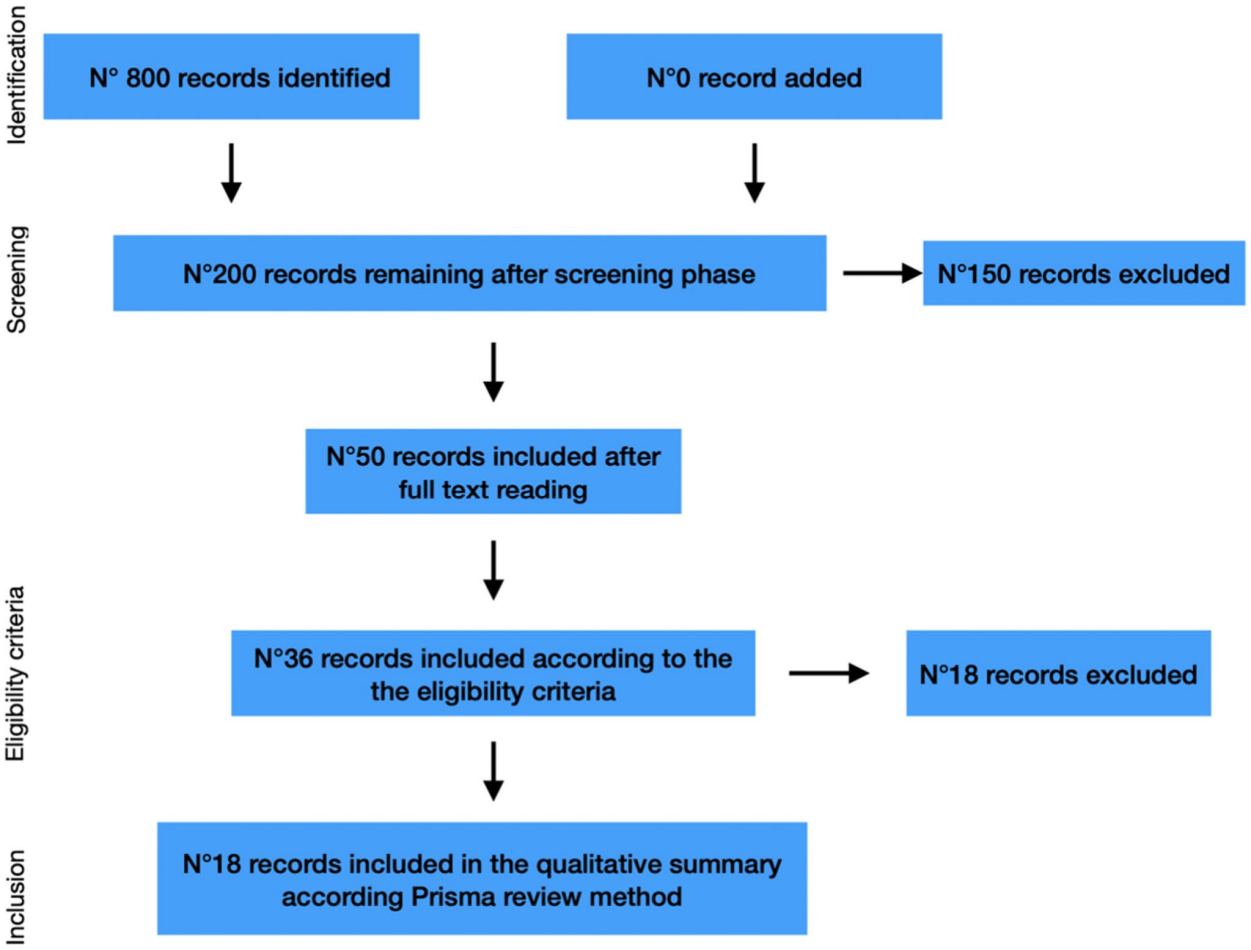
Flow chart.

This process is graphically described in Figure 1.

### Characteristics of the sources of evidence

For each of the included studies, a summary was made of the type of settings, objective, sample, data analyzed, techniques used, and conclusions. In order to make this summary usable, a special table was constructed containing the items below and is given in full in the Appendix.

### Results of individual sources of evidence

The studies selected for this review were grouped according to the particular method of investigation or description of personality used. Approximately four broad categories were thus identified: articles focused on the bodily and behavioral element; articles based on semantic analysis of the self-descriptions provided; articles referring the study of personality to an integrative theoretical background; and articles emphasizing the use of machine learning methods. All articles refer to trait theory as the prevailing epistemological background.

Analysis of the selected studies pertaining to the first category revealed the existence of a number of trends regarding the attribution of personality traits or personality analysis as a function of bodily elements. In a first article (Hu et al., 2018) reviewed, the general disposition of people to form a consistent and reliable structure of trait considerations from body shapes was measured. It was thus scientifically proven how a numerous variety of personality traits are inferred from body shapes, and how these personality inferences are deeply related to the physical characteristics of the body shapes themselves (Iennaco et al., 2020). Moreover, they reflect the nuanced personality characteristics related to the Big Five domains of extroversion, conscientiousness and agreeableness. In another article (Berkovsky et al., 2019), on the other hand, the question of the possibility of delineating personality through the use of physiological responses to external stimuli was raised, on the assumption that they are not consciously controllable and thus as such can be defined as reliable indicators of emotional reactions attributable to personality traits. The research used images with affective valence and video stimuli, as well as eye-tracking data, and upon analysis of the results, the study found that seven personality traits (predicting an addition of two traits to those proposed by the Big Five model) were predictable with more than 90 percent accuracy. An additional article in this category focused on finding associations between facial morphology and personality traits. It was established that humans are able to perceive with a certain degree of accuracy certain personality traits from faces, even if only photographed. For example, facial symmetry is predictive of extroversion (Pound et al., 2007), while the width-to-height ratio of the face has been found to be related to the presence of various traits, such as: dominance, commitment to achievement, deception, risk-taking, and aggression (Carre and McCormick, 2008; Valentine et al., 2014). The study thus gathered new evidence validating the hypothesis that personality is related to facial appearance. The results specifically showed that real-life photographs taken in natural situations can predict personality traits through the use of computer vision algorithms. In particular, conscientiousness was found to be more easily recognized than the other four traits. Finally, an additional paper (Kabbara et al., 2020), included in this category, starts with scientific evidence pertaining to the long-identified neural substrates underlying personality. The innovative contribution of the study started from the consideration that much of the understanding of personality-related differences in functional connectivity has always been studied through stationary analysis, which does not seem to be able to capture the complex dynamic properties of brain networks. In the article, however, it is hypothesized that the study of the dynamic properties of brain network reconfiguration over time may lead to new insights into the neural substrate of personality through the study on a millisecond time scale, of the dynamics of brain connectivity patterns, performed using electroencephalography (EEG) and magneto-encephalography (MEG).

An original article pertaining to the category of observing personality as a function of manifest behavior is that of Tekofsky et al. (2013) in which the potential offered for assessment by a particular type of behavior was investigated: playing video games. The specific research question in this case concerned the investigation about the possible correlation existing between gaming style and personality, and the authors performed a series of operations aimed at ensuring scientificity of the data. In fact, they significantly assessed personality data, through the Big Five questionnaire; quantified playing style, through the choice of a game whose statistics were accessible and descriptive data and that detailed it by making explicit the player’s choices and performance. Finally, the authors took into account recruiting a sufficient number of participants by marketing the research and creating a dedicated website to collect the results. Analysis of the collected data showed that in general, playing style is significantly correlated with personality, with many correlations reaching an effect size greater than 1. The correlations were broken down into three main items: the Unlock Score per Second playing variable was found to be strongly correlated with personality, especially with the Conscientiousness and Extroversion item; conscientiousness and speed of action correlated negatively as did work ethic with performance at the game.

As for the second group of articles, which includes those based on semantic analysis of self-descriptions provided in various modalities, first of all on social but also in generically produced texts, a fairly large scientific production was found starting from the consideration of the dissemination through short and simple messages of emotional states, thoughts and feelings by the millions of users of the global social network. Such messages constitute a rich source of data that, if scientifically analyzed, are able to provide substantial information about personality profiles. The first article (Park et al., 2015) in fact investigates and evaluates the possibility of arriving at a description of personality based on the language used in social media. It starts from the consideration of previous studies carried out in this field based on the so-called “closed vocabulary” method (Schwartz et al., 2013) that starts from lists of words grouped into categories, counting their frequencies and thus analyzing the messages written in social to predict users’ personality traits. As an alternative to this method, the authors describe the open vocabulary procedure in which the linguistic sample is defined by single, non-categorized words; by semantically connected phrases and groups of words; and, finally, by nonverbal symbols (e.g., emoticons, punctuation). This method has been shown to be better at describing personality types in a richer way, highlighting individual differences. The authors therefore proceed by questioning the ability of this procedure, called language-based assessments, to define itself as a new modality for personality assessment, offering itself as a viable alternative to self-report questionnaires. Following analysis of the results, they concluded that the method investigated possesses numerous advantages: it is quick and inexpensive, avoids some of the biases present in self-report questionnaires, and shows a good degree of agreement with the conclusions reached by such questionnaires. In addition, more accurate information can be achieved than that obtained from individual observer reports.

A similar study (Fernandez et al., 2021) focuses on determining how social LinkedIn can convey accurate personality information. The study postulates that individuals through a variety of indicators have an interest in reporting their personality traits on the social, and from the analysis of the results, some 33 LinkedIn indicators were identified as being considered signals of personality traits. To cite some of them as examples it is reported that choosing to use an artistic photo as a background, being able to speak multiple languages, having taken part in artistic activities, or listing the creative skills possessed are defined as indicators of openness to experience; while possessing an up-to-date profile indicating the most recent work experience and a short resume are considered to be indicators of conscientiousness. This study thus demonstrated the veracity of personality signals inferred through LinkedIn, situating this assertion within the frame of reference given by signal theory (Bangerter et al., 2012), which considers that the more accurate the information, the more it requires signals that are costly (in that they take a lot of time or effort to emit) and difficult to alter.

The study by Neuman and Cohen (2014) likewise is based on a new semantic vector approach (Turney and Pantel, 2010) to personality assessment and, through the construction of vectors representing personality dimensions and disorders, measures the similarity between these and texts written by people. Vector semantic models indicate that the meaning of a word, and the concept it represents, can be identified by analyzing the words that appear in correlation with the target word in a given context. The approach proposes representing the relevant words in a sentence as a vector in a semantic space and measuring the distance between the vector composed of these words and the vector of words representing a personality trait. The closer two vectors are the more similar, the sentence is to the personality vector and, therefore, this gives confidence to the hypothesis that the sentence represents the trait. Thus, the degree to which the trait is considered to be represented in the text is defined by the similarity score obtained. Following their analysis, the authors concluded that the results show agreement with the meta-analysis that examined the relationships between the five major factors and personality. Although this agreement provided empirical support for the approach, it was not considered by them as a definitive validation but only as a first step in providing empirical support to reach the minimum level of validity.

With reference to personality analysis based on trait detection, described by the Big Five, from nonverbal auditory and visual cues extracted from brief (30–120 s) self-presentations, Batrinca et al. (2011) investigate the effectiveness of some 29 traits possessed by such cues. The conceptual assumption guiding their investigation is that of “thin slices” (Ambady and Rosenthal, 1992) which refers to the amount of short expressive behaviors on the basis of which humans generate very accurate judgments about the personality characteristics of an individual or group. The results of the study showed that the easiest traits to be automatically detected during self-presentation were found to be conscientiousness and emotional stability, and the authors provide explanation for the phenomenon by stating that the former trait turns out to be related to involvement in task-related behavior while the latter to the emotional reactions it elicits; however, this is not the case for the dispositions of agreeableness and extroversion, which are not activated entirely. Although limited, the results the study arrives at seem to take a first step toward the development of automatic systems to aid in personality detection. The penultimate macro group of articles refers to the idea of the need to use an integrated type of theoretical background that expands the study perspective traditionally related to the topic by introducing the consideration of previously unexamined factors. In the first of them (Dunlop, 2015) the author, Dunlop, aimed to extend the perspective hitherto used by personality psychology with respect to the perception of personality variation within the different social roles held by a person. The contextualized approach as currently considered, according to the author, while interesting shows itself to have a limitation because it is based, almost exclusively, on the assessment of personality traits, which are certainly configured as important components but nevertheless appear to be inadequately representative of all aspects of the person. The article therefore first delves into the nature of such relevant personality traits and argues that just as the recognition of three conceptual levels, namely traits, goals and life narratives, have proved useful within general theories of personality, similarly they could serve a similar function in contextualized approaches to personality by broadening their perspectives of observation. The author illustrates scientific evidence in particular of the predictive capacity of context-specific goals and narratives by adopting a relational meta-theory in the study of personality, with a significant improvement in the understanding of personality through combining context assessment with a multilevel conception of personality. Highlighting the very concept of contextualized personality is necessary because the measurement of personality characteristics cannot be conceived outside the inevitable influences that the environments, in which these characteristics are assessed, exert on it.

McAbee and Connelly (2016) start from the general consideration of how the study of personality, historically, has always been affected by a sharp contrast between the element of accuracy and error in the judgments made about it. The authors have referred both to research that supports the thesis of accuracy and thus stability of judgments over time (Roberts and DelVecchio, 2000) and to studies that have instead identified consistent errors of judgment (Malle, 2006) such that in the 1980s the real existence and concreteness of the construct of personality traits was questioned. They state that while the consensus that is established among researchers turns out to increase as a function of the greater accuracy of trait judgments the same phenomenon does not occur, in a mirror-image fashion, when errors of perception are recorded that, instead, make the consensus more discrepant: while much is known about the conditions under which evaluators agree on personality trait judgments less is known about the outcomes associated with disagreement. The reached conclusion of the authors is that experimental designs with many evaluators are hardly used in the study of trait etiology and outcomes to distinguish between shared and unique perceptions, and for the purpose of separating these perceptions, they propose the Trait-Reputation-Identity (TRI) model in which they illustrate the tools of consensus and uniqueness. Specifically, in the model, Trait is considered a general factor that captures the common variance in self-reports and observers; Identity, on the other hand, captures the variance in self-reports not shared with observers; finally, Reputation captures the residual variance in observers’ reports, resulting from both errors in perceptions and information relevant to the trait but not available *per se*. In the authors’ intentions, then, the Trait-Reputation-Identity Model can offer a unified approach in the study of individual differences in personality traits, unique self-perceptions and personal reputation. As part of the expansion of personality observation modalities based on trait theory Roberts (2018) in his article proposes a revision as an integrative function of his own Socio-genomic model of personality traits that originally started from three essential assumptions: the acquisition, thanks to advances in the field of biological research, of a dynamic notion of DNA through which it became clear that a person’s fate is not written in the “code” in an ineluctable way and that DNA expression can be modified as a function of lived experiences; the importance of states and the relationship between traits, states, environments and biological factors and finally the acquisition of the importance of considering, in the study of personality traits, also observations inferred from the behavioral characteristics of non-human species since the assumption that the genome is conserved across species could explain the nature of some human personality traits as a function of animal personality traits. As in the original theorization, within the revised version, states and traits are found to be correlated and environments are considered to be at the origin of variations in states but the new model introduces, in addition to these, epigenetic mechanisms that determine flexible and elastic systems, which are at the origin of traits.

So, traits are based on DNA (or fixed factors), flexible systems, elastic systems, and state fluctuations. Epigenetic systems determine the change in traits; changes in epigenetic systems, on the other hand, lead to changes in states that are, however, only apparent since they are mediated by the change in traits. According to the author, the current system of studying and observing personality (tests used by professionals) cannot accurately capture the percentage of “influence” of flexible, elastic systems, fixed factors and state fluctuations in trait configuration because it assumes that a typical personality measure must capture the fixed aspect of personality that would determine future outcomes. But this is not a tenable assumption according to socio-genomic conceptualization, and in the author’s intentions the identification of flexible and elastic systems would imply concrete patterns of change, and lack of change, within phenotypes such as personality traits.

A further study selected for the topic, starts from the consideration of the need for a new assessment model. Wright et al. (2019) substantiates the assertion by clarifying how psychological assessment grounds personality patterns on nomothetic principles thus rooting itself in patterns of individual differences and to ascertain an individual’s position relative to others grounds the comparison on one or more dimensions of functioning through normative distributions. This mode, however, produces an incomplete picture of the processes affecting an individual, for two main reasons: because the same model is applied to all in a static form, underestimating the dynamic element inherent in behavior, and because personality structure is not entirely reducible to the set of behavioral elements, but must include the associations existing between them and environmental characteristics. To this end, the author then introduces group iterative multiple model estimation (GIMME) (Gates and Molenaar, 2012), which is useful for conducting multivariate search of the pattern of associations between intensively sampled data. GIMME is based on a unified structural equation modeling framework that provides estimates for both lagged and contemporaneous effects and is a link between two useful methods for analyzing time series data: vector autoregression (VAR) and structural equation modeling. In conclusion, the authors state that GIMME offers interesting study options in dynamic personality assessment while cross-sectional personality assessments fail to capture the dynamics that are supposed to originate traits. The aim of the study, however, was not to reach definitive conclusions about general personality processes; rather, to highlight how the model could be used to study the heterogeneity of processes of individuals with the same trait or symptom profiles.

Another article focused on the need to rethink the personality assessment procedure in a broader key is that of Mayer (2020) in which the author, reflects on the large amount of data that the clinician collects in the course of the assessment, using and mastering technical languages of different nature, which need useful integrations in order to be able to answer the question of diagnosis. To organize all the data, clarify potential gaps in them, and reduce possible confusion resulting from the necessary merging of multiple theoretical backgrounds of reference, integrative methodologies are often used across theoretical approaches (Blais and Hopwood, 2017) in order to keep the focus on the person rather than on the controversies of the field. The author therefore presents an extension of the Personality Systems Framework for Assessment (PSF-A) originally introduced by Mayer (1993, 1998) to support the assessment process by organizing information about an individual’s background and demographics and personality functions and allowing clinicians to focus on their characterization. The part on organizing contextual information is based on the main areas affecting personality and provides a method for recording general medical information, physical living environments, situations experienced, and social group memberships.

The functional type part, on the other hand, provides a system for recording data about a person’s internal mental life and performance in the broad areas of energy development, knowledge guidance, action implementation, and executive management. The scheme thus proposes a broad view of the client by capturing contextual and personal elements of the client; organizing the technical languages of psychiatric symptoms, personality traits and test scores into a single chart based on personality areas; and identifying personality areas not adequately assessed.

One article included in the selection that is proposed as a real disarticulation of the epistemological perspective outlined so far, based on the enrichment of trait theory, is that of Hogan and Foster (2016) who propose a total revision in a critical key of the study approach traditionally adopted by personality psychology. In that, in fact, the two focuses on which personality science rests are questioned and then an epistemological alternative to them is proposed. The article starts with a strong critique about clinical psychology as centering almost exclusively on psychopathology. In this way, according to the authors, it steers the reading and diagnosis of personality predominantly in a direction they believe is wrong, centered on the generalization that all people are neurotic and that the essential problem is to overcome neurosis. This predominantly psychopathological reading of the complexity of the human being is highly reductive, as it does not allow for an understanding of the many psychological and personality nuances and thus a broader framing of the subject and his or her problems in real life. The same highly critical approach is used for trait theory whose self-referentiality the authors are interested in showing. They in fact, following a bold thought process, assert that the theory seems to be geared, with its procedures and epistemology, to the generalization that all people have traits and that the most important element is to discover these traits, likening this process to that for which a person would undergo a genetic analysis to discover that he or she has genes. They also refute the assertion that traits are true neuropsychological entities by asserting their unfoundedness, and finally they point out that trait theory confuses prediction with explanation, in that if one identifies consistent patterns of behavior by calling them traits, it is not logically possible to then explain these same patterns in terms of traits. To understand human nature, therefore, the authors present an alternative based on evolutionary and interpersonal theory. Interpersonal theory asserts that social interaction is what allows coherence and logic to be given to what happens in life: all the contents of an individual’s consciousness are the result of interactions (past and present) and these guide action in the world. How people behave with others provides the data for interpersonal theory.

Finally, in the macro category of articles highlighting the use of machine learning methods, four studies were counted in which the use of devices is made for automatic recognition of personality traits (Alam and Riccardi, 2014) from the analysis of language, visual expressions or textual content with the goal of constructing classifiers through a supervised machine learning approach, which learns patterns from the data. In the first one (Sperandeo et al., 2019), the authors analyze verbal and nonverbal behavior through an approach directed at automatically recognizing personality traits using a collection of video blogs (vlogs) selected from Youtube, in which a person speaks while looking at the camera showing face and shoulders and vloggers illustrate a product or tell about an event. Through Amazon Mechanical Turk (an Amazon suite that enables computer programmers to coordinate human intelligences to perform tasks that computers cannot), the annotation of the vloggers’ personality traits was defined, along with their automatic transcription; audiovisual features included in the data set were analyzed along with lexical, psycholinguistic, and emotional features extracted from the transcript. From the observation and analysis of the data obtained, the authors derived the assumption that the performance of each trait varies for different defined feature sets, so the same feature set or architecture may not be effective for the prediction and analysis of all personality traits.

Another paper (Youyou et al., 2015) included in this category focuses on comparing the accuracy of personality judgments made by humans and artificial intelligence (Kosinski et al., 2013) using a sample of 86,220 volunteers who responded to a personality questionnaire. The premise of this study starts from the consideration of the fact that although it is still believed that accurate personality perceptions are possible only due to the capabilities of the human brain, current developments in machine learning models have shown that computer models are also capable of making appreciable personality judgments by analyzing and evaluating digital records of human behavior. The basic theoretical assumption used by the authors in the study was that of the realist approach, which assumes that personality traits represent real individual characteristics and measures the accuracy of personality judgments through the criteria of agreement between selves, between judges, and through external validity. Personality judgments obtained from the computer were based on Likes posted on Facebook, of which their predictive ability of both personality and other psychological traits has previously been demonstrated (Sperandeo et al., 2021). Judgments from humans, on the other hand, were obtained from the Facebook contacts of participants, who were asked to describe them using a 10-item version of the IPIP personality measure. Three types of final considerations emerged from the data: computer predictions based on a generic digital judgment (“Facebook likes”) are more accurate than those obtained from participants’ Facebook friends; computer models exhibit greater agreement among judges; and computer-generated personality judgments have greater external validity in cases of substance abuse, political attitudes, and physical health. Therefore, the authors conclude by stating that, because of their research findings, personality can be adequately predicted and described by computer systems.

### Conclusion

Personality assessments have historically been conducted from scientific materials, tests and questionnaires, specially created and standardized according to the population under investigation, aimed at highlighting the presence of certain specific patterns of behavioral modes of response to the environment. However, thanks to research and observational studies, it has become common knowledge that personality manifests itself in many subtle ways that often, said standardized materials fail to capture. Thanks to the “big data” leaning, researchers have focused their attention on highlighting points of observation tangential to traditional ones that can offer assessments of personality from broader cues and thus capture more of the complexity of the person. This review is presented as an initial attempt to survey the production in the literature with respect to the topic and had as its main purpose to highlight how the use of observation models based on aspects previously considered scientifically uninformative (body, linguistic expression, environment) with respect to personality analysis proves to be a valuable resource for drawing up personality profiles that are more comprehensive and capable of capturing more of the complexity of the person. What has emerged is a rapidly expanding field of study because of the need to continue to identify rich and in-depth elements of interpretation that will enable us to grasp the continuous evolutions that human personality undergoes, depending on the ever-changing and uncertain conditions of the ecosystem in which life is structured and the increasingly complex challenges it poses.

## Author contributions

LM, GC, NL, EG, CG, and NM designed the research design. EM, OR, and VR contributed to the selection of sources. CS, RS, and DC contributed to proofreading. All authors contributed to the article and approved the submitted version.

## Conflict of interest

The authors declare that the research was conducted in the absence of any commercial or financial relationships that could be construed as a potential conflict of interest.

## Publisher’s note

All claims expressed in this article are solely those of the authors and do not necessarily represent those of their affiliated organizations, or those of the publisher, the editors and the reviewers. Any product that may be evaluated in this article, or claim that may be made by its manufacturer, is not guaranteed or endorsed by the publisher.
