# Supplementary material for: A scoping review on innovative methods for personality observation
Source: Front Psychol. 2023 Mar 8;14:1112287. doi: 10.3389/fpsyg.2023.1112287 (PMC10031124; doi:10.3389/fpsyg.2023.1112287)
Supplement: Supplementary file 1 [file Table_1.docx]

| **Title** | **Year** | **First author** | **Study aim** | **Sample** | **Data analysed** | **Technique** | **Conclusions** |
| --- | --- | --- | --- | --- | --- | --- | --- |
| 1. A Multi-Rater Framework for Studying Personality: The Trait-Reputation-Identity Model | 2016 | Samuel T. McAbee | To present a new model (TRI model) that separates personality variance into consensus about underlying traits (Trait), unique self-perceptions (Identity), and impressions conveyed to others that are distinct from self-perceptions (Reputation). | N= 478 (targets)  N = 1,434 (observer) | Self-report data were available 478 participants (targets) who completed the Big Five Inventory, collected during the fall of 1998. Three observers (N = 1,434) rated each of these 478 targets using the observer-report version of the BFI. | Latent variable models delineating shared versus unique rater variance across item ratings from one self-report and three observer-reports for each Big Five trait, respectively. | Researchers can use the TRI Model to achieve a more sophisticated view of personality’s impact on life outcomes, developmental trajectories, genetic origins, person–situation interactions, and stereotyped judgments. |
| 2. Brain network dynamics correlates with personality traits | 2019 | Aya Kabbara | To evaluate the feasibility of using dynamic network measures to predict personality traits | A total of 56 healthy subjects were recruited (29 women). The mean age was 34.7 years old (*SD* = 9.1 years, range = 18–55). | It was tested the hypothesis on two datasets: 1) Resting‐state EEG data acquired from 56 subjects, and 2) Resting‐state MEG data provided from the publicly available Human Connectome Project (HCP) MEG2 release including 61 subjects. | Using the EEG/MEG source connectivity method combined with a sliding window approach, dynamic functional brain networks were reconstructed from two datasets: 1) Resting state EEG data acquired from 56 subjects. 2) Resting state MEG data provided from the Human Connectome Project. Then, several dynamic functional connectivity metrics were evaluated. | These findings highlight the importance of tracking the dynamics of functional brain networks to improve our understanding about the neural substrates of personality. |
| 3. First Impressions of Personality Traits From Body Shapes | 2018 | Ying Hu | To explore personality inferences made from body shapes. | (*N* = 76; 17 men; age: *M* = 20.40 years, *SD* = 2.89) | Undergraduate students (*N* = 76; 17 men; age: *M* = 20.40 years, *SD* = 2.89) rated body shapes for personality traits.  The stimuli were 140 (70 female, 70 male) bodies generated randomly using the skinned multiperson linear (SMPL) model | Skinned multiperson linear (SMPL) model | Personality-trait profiles were predicted reliably from a subset of the body-shape features used to specify the three-dimensional bodies. Body features related to extraversion and conscientiousness were predicted with the highest consensus, followed by openness traits. This study provides the first comprehensive look at the range, diversity, and reliability of personality inferences that people make from body shapes. |
| 4. Automatic Personality Assessment Through Social Media Language | 2014 | Gregory Park | Language use is a psychologically rich, stable individual difference with well-established correlations to personality. This paper describes a method for assessing personality using an open-vocabulary analysis of language from social media. | A subset of myPersonality users (N 71,556) a third- party application on the Facebook social network | Every status message written by study volunteers between January 2009 and November 2011, totaling over 15 million messages. Users wrote an average of 4,107 words across all status messages. Measures of personality traits as defined by the NEO-PI-R five factor model. A subset of users (n 348) completed an additional 336-item IPIP proxy to the NEO-PI-R, designed to assess 30 facet-level personality traits. | Language based assessments (LBAs) capable of capturing true personality variance.  Mono-trait correlations (Pearson rs); z test for independent correlation coefficients. | Social media language is rich in psychological content and can be leveraged to create a fast, valid, and stable personality assessment. Comparisons with informant reports and external criteria suggested that language based assessments (LBAs) are capable of capturing true personality variance. Predictions were stable over time, with test–retest correlations on par with self- report questionnaires of personality. |
| 5. A Vectorial Semantics Approach to Personality Assessment | 2014 | Yair Neuman | Computational personality is a new field that involves the automatic classification of individuals’ personality traits that can be compared against gold-standard labels. In this context, we introduce a new vectorial semantics approach to personality assessment, which involves the construction of vectors representing personality dimensions and disorders, and the automatic measurements of the similarity between these vectors and texts written by human subjects. | A corpus of 2468 essays written by students. | Each essay was analyzed through a Part-of-Speech tagger and only nouns, verbs, adjectives, and adverbs were processed for further analysis. Next, it was measured the similarity between each of the essays and each of the PDs vectors by using the term-to-context matrix developed by Turney | Monte Carlo Estimate; Classification and Regression Tree (CRT) model; rotation estimation | The vectorial semantics approach merges the idea of VSM with personality assessment. This is a novel approach that is different from the other approaches used for automatic personality assessment. Using this approach for measuring the level of each PD in a text, the results largely agree with the most comprehensive meta-analysis that examined the relationships between the big five factors and PDs. While this agreement provides empirical support for the approach, it cannot be considered as a final validation but only as a first and primary step in providing empirical support for a minimal level of validity. |
| 6. Detecting Personality Traits Using Eye-Tracking Data | 2019 | Shlomo Berkovsky | To propose a framework for objective personality detection that leverages humans’ physiological responses to external stimuli. The rich modality, increasing accuracy, and decreasing costs of modern sensing technologies pave the way for their deployment in a range of real-life applications. We propose to use such technologies for capturing physiological signals, e.g., brain activity, eye saccades, or skin conductivity, produced by the human body in response to stimuli. As many of these signals are bodily responses that cannot be consciously controlled, we posit that they can be considered as reliable and valid indicators of the human’s reaction to the stimuli and to the emotions evoked by the stimuli, which we attribute to the personality traits. In this work we set out to study whether observable patterns in physiological responses to stimuli can serve as predictors of personality traits. | 21 subjects, students or staff of a research organization, were recruited. 18 subjects were aged 18 to 30 whereas the other 3 were older than 30 | A subset of images from the International Affective Picture System (IAPS) dataset were used. This is a well- studied dataset, where each image is assigned scores corresponding to different emotions. Plus, videos were used from the English version of the FilmStim dataset. Video stimuli representing seven emotion types – fear, tenderness, anger, neutral, sadness, amusement, and disgust – were selected based on their pre-annotated arousal-valence scores.  The subject’s physiological responses to the stimuli were captured by the SMI ETG | Standard implementations of Weka, an open-source data mining toolbox. Specifically, seven classifiers were deployed: AdaBoost (AB), Decision Tree (DT), Logistic Regression (LR), Naive Bayes (NB), Random Forest (RF), Support Vector Machine (SVM), and k- Nearest Neighbour (kNN | In this work it was developed a framework for predicting human personality traits using physiological responses to external stimuli. It was found that the Naive Bayes algorithm, in conjunct- tion with feature selection, substantially outperformed other machine learning algorithms. Seven traits were predicted with accuracies greater than 90%. Comparing the image and video stimuli, we found that the latter performed better, while their combination improved the predictive accuracy. |
| 7. Contextualized Personality, Beyond Traits | 2015 | William l. Dunlop | Personality psychologists have become increasingly interested in how personality varies across social roles. Within this ‘contextualized’ approach, researchers almost invariably focus on assessing personality traits. Although these characteristics are no doubt important components of personality, there are many aspects of the person that are not adequately represented by traits. This article fleshes out the nature of these additional personality characteristics relevant to contextualized personality. | Theoretical |  |  | Generalized personality is commonly conceptualized to include two additional ‘levels’ beyond traits, manifest in terms of goals and life narratives. Acknowledging these additional levels has offered more complete pictures of certain psychological processes and personality–outcome associations. The consideration of goals and life narratives may inform understanding of personality in its contextualized forms too. Adopting a relational meta-theory, it was argued that traits, goals and life narratives are mutually constituted and that understanding of one of these personality levels requires assessment of all. |
| 8. An Integrated Approach to Personality Assessment Based on the Personality Systems Framework | 2019 | John D. Mayer | This work describes an extension of the personality systems framework to assessment, referred to here as the Personality Systems Framework for Assessment (PSF–A), that builds on Blais and Hopwood’s (2017) recent contribution. | Theoretical |  |  | Using a good system for organizing knowledge during the assessment process reduces the clinicians’ cognitive load, freeing professionals to focus on optimally conceptualizing their clients’ characteristics in addressing the key assessment questions at hand. |
| 9. Focusing Personality Assessment on the Person: Modeling General, Shared, and Person Specific Processes in Personality and Psychopathology | 2019 | Aidan G.C. Wright | to present a method, Group Iterative Multiple Model Estimation (GIMME) for simultaneously studying general, shared (i.e., in subgroups), and person-specific processes in intensive longitudinal behavioral data. | 116 participants attended the baseline assessment for the daily diary study and were enrolled in a 100-day daily diary protocol. the effective sample size for the GIMME analyses was n = 94 | Daily affect was measured using a subset of Positive and Negative Affect Schedule (PANAS) items. *Daily Interpersonal Behavior.* Daily interpersonal behavior was measured using a subset of the Interpersonal Adjective Scales items. Daily stress was measured using a self-report version of the Daily Inventory of Stressful Events Difficulties with daily functioning were assessed using a single item, referenced to the last 24-hours. | All GIMME analyses described here were conducted using the ‘gimmeSEM’ function provided by the *gimme* R package | The study of personality and psychopathology, and therefore their assessment, must move beyond a nomothetic approach, whereby individuals scores are only understood as relative to others. GIMME provides a way forward, by searching for general, shared, and person-specific contemporaneous and lagged associations in intensive data. Thus, it can be used to build generalizable models from the bottom up |
| 10. PsyOps:  Personality Assessment Through Gaming Behavior | 2013 | Shoshannah Tekofsky | To determine whether video games are a valuable addition to the arse- nal of personality assessment methods | 13,376 Battlefield 3 players | Data analysis progressed in five steps.  1. Creating and applying integrity filters to the data set.  2. Determining play style based on game statistics.  3. Determining personality based on IPIP scores.  4. Defining relevant (sub)samples.  5. Calculating correlations between play style and personality for all (sub)samples. |  | We found that play style and personality do correlate significantly, showing three key themes. Con- scientiousness is negatively correlated with speed of action. The game variable Unlock Score per Second correlates most often and most strongly with personality, especially with Conscientiousness and Extraversion. Work ethic correlates negatively with performance in the game. Apart from these three themes, subsamples differ in correlational patterns. An additional result was found when performing a post- hoc analysis on age. Correlations between age and play style were greater than those between play style and personality. |
| 11. Please, Tell Me About Yourself: Automatic Personality Assessment Using Short Self-Presentations | 2011 | Ligia Maria Batrinca | This paper addresses the automatically detection of the Big Five personality traits from short (30-120 seconds) self-presentations, by investigating the effectiveness of 29 simple acoustic and visual non-verbal features. | 89 participants: 46 male and 43 female 47 young people, i.e. under 25, and 42 adults, i.e. over 25 | The Big Five scores were calculated summing single raw scores, properly inverted, per each personality trait. The analysis was conducted by means of a series of ANOVA with dependent variables, the factorial scores for Extraversion, Agreeableness, Conscientiousness, Emotional Stability and Creativity, and Age and Gender as predictors. The (linear) relationships between our features and the Big Five personality traits were analyzed by means of number of backward linear regression analyses, one per each trait, with all our features as predictors. | we applied the Weka implementation of the Support Vector Machine Recursive Feature Elimination (SVM-RFE) algorithm (called Support V ector Machine attribute evaluation method in Weka) in order to evaluate the importance of a feature.  A linear SVM is a hyper-plane that separates two classes of examples (positive and negative) maximizing the separation margin | Our results show that Conscientiousness and Emotional Stability/Neuroticism are the best recognizable traits. The lower accuracy levels for Extraversion and Agreeableness are explained through the interaction between situational characteristics and the differential activation of the behavioral dispositions underlying those traits. |
| 12. Predicting Personality Traits using Multimodal Information | 2014 | Firoj Alam | In this study, we present an approach to automatically recognize personality traits using a video-blog (vlog) corpus, consisting of transcription and extracted audio-visual features. We analyzed linguistic, psycholinguistic and emotional features in addition to the audio-visual features provided with the dataset. We also studied whether we can better predict a trait by identifying other traits. | Systems are required to recognize “Big-5” personality traits from Youtube and/or Mobile datasets.  It contains 348 training, 56 test instances, consisting of 404 vlogs in total, where 194 (48%) are male and 210 (52%) are female vloggers. | For the study, we experimented with audio-visual features that had been released with the dataset and also extracted lexical, POS, psycholinguistic and emotional features from the transcription | We generated our classification models using Sequential Minimal Optimization (SMO) for Support Vector Machine (SVM) for each feature set as described above. We used different kernels for different feature sets, such as linear kernel for lexical (Lex) and POS features and polynomial kernel for audio-visual (AV), psycholinguistic (LIWC), emotional (Emo) and traits (Traits) features. | We obtained very promising results compared to the official baseline. Performance of the model using emotional feature set is very low compared to the other feature sets, however, it helps in combination. We plan to experiment with the traits and emotional features with other datasets in the future. |
| 13.Using available signals on LinkedIn for personality assessment | 2021 | Sebastien Fernandez | To determine if LinkedIn profiles convey accurate information about in- dividuals’ personality traits. Drawing from signaling theory, we expect that individuals portray themselves in a manner that will reflect their personality. | Graduates (N=607) from a hospitality management school in Switzerland (60.5% of them are women; Age, M = 24.45; SD = 1.63 with the youngest participant being 22 years old and the oldest being 35 years old) | 607 LinkedIn profiles were coded on 33 indicators. |  | The aim of the study was to test to which extent LinkedIn offers accurate information about personality. We identified 33 LinkedIn indicators to serve as signals of personality traits. The results demonstrated that most of these indicators provide signals of the expected personality traits. |
| 14.Focusing Personality Assessment on the Person: Modeling General, Shared, and Person Specific Processes in Personality and Psychopathology | 2019 | Aidan G.C. Wright | Personality and psychopathology are composed of dynamic and interactive processes  among diverse psychological systems, manifesting over time and in response to an individual’s natural environment. Ambulatory assessment techniques promise to revolutionize assessment practices by allowing access to the dynamic data necessary to study these processes directly. Assessing manifestations of personality and psychopathology naturalistically in an individual’s own ecology allows for dynamic modeling of key behavioral processes. | Theoretical |  |  | The study of personality and psychopathology, and therefore their assessment, must move beyond a nomothetic approach, whereby individuals scores are only understood as relative to others. With the rapid development of ubiquitous computing and ambulatory assessment techniques, intensive sampling of behavior can now be used to develop personalized models of personality. |
| 15.Assessing the Big five personality  traits using real-life static facial  images | 2020 | Alexander Kachur | To present new findings demonstrating the statistically significant prediction of a wider set of personality features (all the Big five personality traits) for both men and women using real-life static facial images. | Volunteer participants (n = 12,447) provided their face photographs (31,367 images) | A self-report measure of the Big Five traits | We trained a cascade of artificial neural networks (ANNs) on a large labelled dataset to predict self-reported Big five scores. the highest correlations between observed and predicted personality scores were found for conscientiousness (0.360 for men and 0.335 for women) and the mean effect size was 0.243, exceeding the results obtained in prior studies using ‘selfies’. | The findings strongly support the possibility of predicting multidimensional personality profiles from static facial images using Anns trained on large labelled datasets. |
| 16.Computer-based personality judgments are more accurate than those made by humans | 2014 | Wu Youyou | To comparee the accuracy of human and computer-based personality judgments | a sample of 86,220 volunteers, who filled in the 100-item International Personality Item Pool (IPIP) Five-Factor Model of personality questionnaire  Computer-based personality judgments, based on Facebook Likes, were obtained for 70,520 participants.  To compute self-other agreement and external validity, we used a sample of 17,622 participants judged by one friend; to calculate interjudge agreement, we used a sample of 14,410 participants | Computer predictions based on a generic digital footprint (Facebook Likes) are more accurate (r = 0.56) than those made by the participants’ Facebook friends using a personality questionnaire (r = 0.49); computer models show higher inter-judge agreement; and computer personality judgments have higher external validity when predicting life outcomes such as substance use, political attitudes, and physical health; for some outcomes, they even outperform the self-rated personality scores. | We used LASSO (Least Absolute Shrinkage and Selection Operator) linear regressions with 10-fold cross-validations, so that judgments for each participant were made using models developed on a different subsample of participants and their Likes. | Computers outpacing humans in personality judgment presents significant opportunities and challenges in the areas of psychological assessment, marketing, and privacy. |
| 17.A Revised Sociogenomic Model of Personality Traits | 2018 | Brent W. Roberts | To update the sociogenomic model of personality traits. Specifically, to outline a broader and more comprehensive theoretical perspective on personality traits than offered in the original version of the sociogenomic model of personality traits. | Theoretical |  |  | The article attempted to update the sociogenomic model of personality traits. The revision is aspirational as the newly identified systems that provide mechanistic explanations for the patterns of growth and change in personality traits may remain beyond the methods of biology research typically employed with non-human animals for years. Nonetheless, the identification and integration of pliable and elastic systems provides answers to questions left unanswered by the original sociogenomic model |
| 18.Rethinking personality | 2016 | Robert Hogan | This paper provides an overview and critique of personality psychology. It discusses personality psychology in terms of three major movements: clinical psychology; trait theory; and interpersonal theory. The paper criti- cizes clinical psychology for focusing on psychopathology, and it criticizes trait theory for being circular. | Theoretical |  |  | This paper provides an alternative, grounded in evolutionary theory and framed in terms of interpersonal processes. Next, it criticizes traditional assessment for trying to measure entities rather than predict outcomes. Finally, the paper re- views standard criticisms of personality assessment (e.g., low validity, faking) and argues that these criticisms lack merit. |
